# Construction of middle-phase microemulsion system and its micro-mechanism on displacing residual oil in low-permeability porous media

**DOI:** 10.3389/fchem.2024.1465706

**Published:** 2024-11-07

**Authors:** Tianjiang Wu, Teng Wang, Yingxue Hu, Jiajun Chen, Junwei Su

**Affiliations:** ^1^ School of Human Settlements and Civil Engineering, Xi’an Jiaotong University, Xi’an, China; ^2^ Oil and Gas Technology Research Insititute of Changqing Oilfield, China National Petroleum Corporation, Xi’an, China

**Keywords:** middle-phase microemulsion, system construction, solubilization capacity, micro-oil displacement mechanism, low-permeability reservoir

## Abstract

The application of medium-phase microemulsion in enhancing oil recovery technology represents a significant area of research, particularly for improving production in low-permeability reservoirs. The oil recovery can be increased to 80%~90%. In order to further improve the recovery rate of low-permeability reservoirs in the late stage of water flooding, a medium-phase microemulsion flooding system was constructed in this paper. The micro-displacement mechanism of the medium-phase microemulsion flooding system was clarified by experimental methods such as phase change and micro-remaining oil distribution. The ability of enhancing oil recovery and the mechanism of increasing oil production were discussed, which provided a basis for establishing a new method of enhancing oil recovery. This study utilizes a mixed surfactant system composed of sodium dodecyl benzene sulfonate and coconut oil fatty acid lipopolyoxyethylene betaine at a mass ratio of 1:3, with n-butanol serving as the cosurfactant. The fish phase diagram was instrumental in determining the critical concentration range for alcohol (1.3%–3.7%) necessary for the formation of middle-phase microemulsions, along with a corresponding surfactant mass concentration of 0.3%–0.7%. Key salinity thresholds for middle-phase formation and disappearance were identified at 1.5% and 6.0%, respectively. Optimal solubilization effects were observed at approximately 4.8% NaCl mass concentration, which effectively reduced interfacial tension to 10^–3^ mN/m. Under specific kinetic conditions, *in-situ* formation of middle-phase microemulsions occurs as surfactants interact with crude oil within reservoir pores. In comparison to traditional water flooding, middle-phase microemulsions enhance viscosity and create an oil wall at the forefront of displacement. This mechanism facilitates the aggregation and movement of residual oil, which is crucial for enhancing crude oil recovery. Moreover, middle-phase microemulsions exhibit strong solubilization capabilities, making them particularly effective for mobilizing oil in blind-end and unswept areas. The ultra-low interfacial tension achieved between the microemulsion and crude oil promotes the elongation and fragmentation of pore-trapped oil into smaller droplets, ultimately aiding in their displacement and recovery via micro-pore outlets. This unique interaction underscores the potential of middle-phase microemulsion flooding to optimize oil recovery processes, especially in challenging reservoir environments such as those encountered in the Changqing Oilfield formations.

## 1 Introduction

After water flooding, residual and remaining oil in the oil layer necessitate tertiary oil recovery to enhance oil extraction efficiency (Arain et al., 2024; Raouf et al., 2024; Tileuberdi and Gussenov, 2024; Youssif and Magda, 2024). Among tertiary recovery methods, chemical flooding stands out as a cost-effective and efficient approach to boost oil recovery, with notable successes observed in China (Olajire, 2014; LIU et al., 2021; Pashapouryeganeh et al., 2022; Cao et al., 2023; Singh and Sharma, 2024; Zamani et al., 2024). Surfactant flooding, polymer flooding, and composite flooding are commonly employed chemical flooding techniques in this domain (Olajire, 2014; Raffa et al., 2016; Afolabi et al., 2022; Li et al., 2023; Jin et al., 2024; Singh and Sharma, 2024; Xu et al., 2024; Kokkinos et al., 2022).

Changqing oilfield, characterized by low porosity, low permeability, strong heterogeneity, and challenges in water flooding (Shang et al., 2015; Wei et al., 2021; YUAN et al., 2021; Yuan et al., 2022), has seen advancements since 2010 with the development of polymer microspheres sized between 50–300 nm to enhance reservoir water flooding practices (Ma et al., 2022; Zhou et al., 2023). Over 20,000 wells have adopted the on-line centralized injection mode via water injection trunk lines, emerging as a primary method for water management and oil stabilization in Changqing oilfield (Wu et al., 2023). As water injection strategies progressed, focusing on the widespread integration of nano-polymer microspheres to optimize water flooding (Gbadamosi et al., 2018; Liu et al., 2022; 2023; Chen et al., 2024), efforts turned towards enhancing oil displacement efficiency. Consequently, middle-phase microemulsion flooding technology (Madhav and Gupta, 2011) has emerged as a novel research avenue for enhanced oil recovery in Changqing’s low permeability reservoirs since 2018, signaling a shift in exploration for improved extraction methods (Santos et al., 2023; Mo et al., 2024; Saboorian-Jooybari and Chen, 2024).

Microemulsions, optically isotropic and thermodynamically stable solutions comprising oil, water, and amphiphilic molecules, exhibit transparency or translucency. These systems can coexist with residual oil, water, or both, categorized by Winsor into Winsor I, Winsor II, and Winsor III microemulsions respectively (Raut et al., 2018; Pal et al., 2023). Winsor III, commonly known as middle-phase microemulsion, represents an optically transparent and thermodynamically stable mixture of oil, water, and surfactant (Santos et al., 2023), first proposed by Schulman and Hoar in 1943 (Hoar and Schulman, 1943) with particle sizes typically ranging from 10–100 nm. Middle-phase microemulsions exhibit potent solubilization capabilities towards oil and water phases, forming ultra-low interfacial tensions that have garnered attention as exceptional oil displacement agents. Enhanced oil recovery mechanisms through microemulsion flooding primarily revolve around achieving ultra-low interfacial tensions due to the presence of surfactants and co-surfactants, effectively reducing interfacial tensions to levels near 10^−3^ mN/m. This process alters rock wettability, decreases capillary pressures, and enables solubilization of both water and oil phases. Winsor I microemulsions efficiently solubilize significant oil quantities, while Winsor II variants dissolve substantial water volumes, particularly addressing oil wax, oil stains, and asphalt insoluble in water. With nanoscale particle sizes, microemulsions can penetrate reservoirs effectively, enhancing oil recovery by displacing remaining oil through intermolecular forces. Their properties like ultra-low interfacial tension, emulsification, solubilization, dispersion, and foaming reduce capillary forces, enhance interfacial wettability, improve flow dynamics, and drive remaining oil extraction. Lowering oil-water interfacial tension via microemulsion application enhances oil washing efficiency and overall recovery rates. Direct observation of mid-phase microemulsion generation in subsurface porous mediums during surfactant-driven processes is crucial for elucidating oil repulsion mechanisms and optimizing repellent fluid properties to boost recovery further.

Microfluidic technology has recently offered insights into the intricate structures of opaque sandstone, enabling direct observation of multiphase flow dynamics within these formations. Unsal et al. (2016) utilized a T-type microfluidic channel integrated with fluorescence imaging to study the *in-situ* generation of mesophase microemulsions. By introducing surfactant solutions and oil phases through separate inlets, effective mixing of aqueous and oil phases occurred within the channel, facilitating clear visualization of microemulsion formation near the oil/water interface using fluorescence microscopy. In quasi-static experiments, significant variations in phase states and physical properties of the microemulsion system were noted concerning mineralization levels and surfactant concentrations, with observations of how these factors influenced *in situ* microemulsion generation within the microfluidic chip. Zhou et al. (2020) leveraging high-precision fluorescence microscopy and microfluidic chip experiments, identified the optimal middle-phase microemulsion formulation under static conditions, aligning with the oil/water interface. Tagavifar et al. (2017) discovered that microemulsions in microfluidic chips establish a persistent boundary layer at initial contact between the surfactant solution and oil, evolving over time due to induced convection. Pulsating Marangoni flow phenomena spontaneously drove microemulsion and surfactant solution into the oil stream, forming sizable emulsion droplets. Yu et al. (2019) investigated the oil displacement mechanisms of Winsor Type I microemulsions within oil-wetted media using an innovative 2.5-dimensional microfluidic chip. Their research highlighted the enhanced recovery potential of Winsor I microemulsions, emphasizing wetting phase propulsion and residual oil solubilization as primary mechanisms. Zhao et al. (2022) developed a specialized microfluidic chip based on a low-permeability core to investigate the efficacy of directly injected microemulsion systems for residual oil displacement post-water flooding. Their findings showcased a 5%–8% improvement in recovery compared to water drive methods, demonstrating the promise of microemulsion applications in enhancing oil recovery processes.

To enhance the oil recovery rate in the late development stages of Changqing oilfield, this study introduces a middle-phase microemulsion flooding system comprising surfactants, alcohols, salts, and other components. Through assessing fundamental properties of the middle-phase microemulsion system and conducting microscopic oil displacement experiments, the microscopic oil displacement mechanism of this system was elucidated. The study delves into its enhanced oil recovery capabilities, discusses crude oil production mechanisms, and lays the groundwork for a novel enhanced oil recovery methodology.

## 2 Experimental procedures

### 2.1 Materials

The chemicals including n-butane, n-hexane, n-octane, and n-decane (≥99.8% analytically pure) were supplied by Shaanxi Changhai Oilfield Additives Co., Ltd.; sodium dodecyl benzene sulfonate (CQ-1) and coconut oil fatty acid polyoxyethylene betaine (CQ-2) (≥90% industrial grade) were supplied by Xi’an Changqing Chemical Group Co., Ltd.; sodium chloride, n-propanol, n-butanol, n-hexanol, and n-heptanol (≥99.8% analytically pure) were supplied by Chengdu Cologne Chemicals Co., Ltd. The simulated oil’s viscosity at 56°C was 2.0 mPa s, sourced from Changqing oilfield. The simulated water used had a salinity of 71637 mg/L, comprising NaCl at 21080 mg/L, CaCl_2_ at 5726 mg/L, and MgCl_2_ at 539 mg/L. The crude oil was supplied from Changqing Oilfield, in which the saturated component accounts for 76.05%, the aromatic component accounts for 17.93%, the resin accounts for 5.55%, and the asphaltene accounts for 0.47%. The wax precipitation point is 34°C, and the density is 0.8331 g/cm^3^.

### 2.2 Experimental method

#### 2.2.1 Construction of microemulsion system

The study involved identifying the diluted phase alkanes present in the simulated oil. Interfacial tension measurements were conducted between n-alkanes of varying carbon chain lengths, crude oil, and surfactants. Alkanes displaying interfacial tensions closest to those of crude oil were selected as equivalent alkanes for further analysis. A screening process was performed to identify an optimal surfactant complex system. This involved adjusting the surfactant ratios to identify a compound system with robust stability and ultra-low interfacial tensions. Additionally, various auxiliary alcohols were investigated to enhance the volume, solubilization capacity, interfacial tension, and stability of the middle-phase microemulsion when combined with the surfactant complex system, leading to the optimization of cosurfactant alcohols.

#### 2.2.2 Evaluation of phase behavior of microemulsion

The experiment involved adding the surfactant compound system and alcohol to plug colorimetric tubes, along with various mass concentrations of NaCl, equivalent volumes of simulated oil, and simulated formation water. Following sealing, the tubes were shaken rigorously over 50 times and left to stand at 56°C for 72 h. The stratification process was observed, and the volume fractions of each layer were measured. A Winsor phase diagram correlating salinity with phase volume fractions was constructed. The solubilization capacity of the microemulsion under varying salinity levels was determined based on the phase diagram, following methods outlined by Huh (1979); Huh (1983).

$$SP_{x}=\frac{V_{x}}{V_{s}}$$ (1)

The Chun-Huh equation is also used to calculate the interfacial tension of microemulsion oil-water phase.

$$\sigma =\frac{C}{SP_{x}^{2}}$$ (2)

In the formula, *SP*_*x*_ is solubilized oil or solubilized water, dimensionless, *V*_x_ is the volume of oil or water entering the microemulsion, mL; *V*_s_ is the volume of surfactant, mL; *σ* is interfacial tension, mN/m; *C* is constant, dimensionless, which can take 0.3.

#### 2.2.3 Microphysical simulation of microemulsion flooding

Microphysical simulation of microemulsions was conducted using a microfluidic chip. The microfluidic pore channel design was derived from the core of Changqing oilfield. Initially, a two-dimensional channel slice was generated using an X-ray micro-computed tomography scanner (Xradia 630 Versa, USA), followed by numerical reconstruction to create the pore network. Subsequently, the microfluidic chip was fabricated via wet etching. Microscopic oil displacement simulations using microemulsions under various injection rates and conditions were performed to investigate the oil displacement mechanism. Two high-precision syringe pumps (CETONI Nemesys S, Germany) were used to accurately control the flow of water and oil through the microfluidic chip. Images of the fluid’s distribution were recorded at intervals of 10–30 s using a digital camera (Canon D800, Japan).

## 3 Results and discussion

### 3.1 Construction of middle phase microemulsion system

#### 3.1.1 Construction of mixed surfactant system

Due to the challenges in observing the color and evaluating stability of the brown crude oil-formed middle-phase microemulsion, experiments were conducted using simulated oil. The formulation of the simulated oil primarily focused on aligning the numerical magnitude of equivalent alkanes between the diluted oil and crude oil to influence microemulsion phase transitions and stability. Interfacial tension measurements between n-butane, n-hexane, n-octane, n-decane, crude oil, and various surfactant systems demonstrated values of 0.1755, 0.0818, 0.0192, and 0.0046 mN/m, respectively. Comparatively, the addition of n-decane led to interfacial tensions closest to crude oil. Hence, n-decane was chosen as the dilution component for the simulated oil, prepared at a volume ratio of crude oil to n-decane of 1:48.

At a simulated formation water salinity of 71,637 mg/L, 56°C, and 4.0% NaCl mass concentration, investigations were conducted on the interfacial tension, solubility, and stability of surfactant CQ-1 and CQ-2 composite systems at varying ratios. Table 1 presents the results, indicating distinct characteristics based on the CQ-1 to CQ-2 mass ratios. Increasing the CQ-1 content led to higher mutual dissolution rates, shorter dissolution times, and gradually decreasing interfacial tensions. When the CQ-1 to CQ-2 mass ratio reached 1:3, the interfacial tension minimized to 0.0049 mN/m, showcasing a transparent solution with excellent stability and absence of layered precipitation after 72 h. Consequently, the optimal mass ratio determined for CQ-1 to CQ-2 was 1:3.

**TABLE 1:** Comparison of properties of middle-phase microemulsions formed by different alcohols.

The mass ratio of CQ-1 and CQ-2	Solubility	Appearance of solution	Interfacial tension (mN/m)	Stability
1:0	—	Clarified solution	0.8505	—
1:1	16 min slow miscible	Turbid solution	0.1890	Layering
1:2	10 min slow miscible	Turbid solution	0.0237	Layering
1:3	5 min rapid miscible	Clarified solution	0.0049	Clear transparent solution
1:4	4 min rapid miscible	Precipitation	0.0076	Precipitation
0:1	—	Clarified solution	0.0632	—

#### 3.1.2 Optimization of middle phase microemulsion additive alcohol

The Hydrophilic-Lipophilic Balance (HLB) plays a crucial role in selecting cosurfactants, with alcohol choice being pivotal in adjusting the HLB value of the middle-phase microemulsion. Alcohol type and molecular weight primarily influence the phase behavior of microemulsions. The addition of high-carbon alcohols significantly impacts the stability of the middle-phase microemulsion, especially in response to temperature variations. The length of the alcohol’s hydrocarbon chain partially dictates the interfacial tension on either side of the interfacial film. Extreme lengths in the hydrocarbon chain can hinder middle-phase microemulsion formation. Excessively long chains result in consistently lower interfacial tensions with the oil phase compared to the water phase. Conversely, overly short chains lead to unequal interfacial tensions on both sides, preventing equilibrium.

Under a fixed 4.0% NaCl concentration, the impact of n-propanol, n-butanol, n-hexanol, and n-heptanol on middle-phase microemulsion formation was studied at surfactant complex system concentrations of 0.1%, 0.3%, and 0.5%. Results detailed in Table 2 reveal that increasing the surfactant concentration allows for achieving ultra-low interfacial tensions with the addition of the specified alcohols. At a 0.3% surfactant concentration, n-propanol and n-butanol yielded interfacial tensions of 0.0061 mN/m and 0.0073 mN/m, respectively. N-hexanol and n-heptanol required a 0.5% surfactant concentration to reach ultra-low interfacial tensions, but failed to form a middle phase. Notably, at a 0.5% surfactant concentration, n-butanol generated an 8.2 mL middle phase volume and solubilized 4.1 mL of oil compared to n-propanol. Consequently, n-butanol emerged as the preferred additive in this context.

**TABLE 2:** Comparison of properties of middle-phase microemulsions formed by different alcohols.

Type of alcholo	Mass concentration of compound system (%)	Volume of middle phase (mL)	Volume of solubilization oil (mL)	Interfacial tension (mN/m)	Stability
n-propanol	0.1	1.3	0.6	0.0243	The middle phase microemulsion disappeared after 20 min
	0.3	2.2	1.1	0.0061	
	0.5	3.1	1.6	0.0062	
n-butyl alcohol	0.1	2.9	1.5	0.0220	The middle phase microemulsion disappeared after 45 min
	0.3	5.7	2.4	0.0073	
	0.5	8.2	4.1	0.0045	
n-hexanol	0.1	No middle-phase microemulsion was formed	—	0.0512	—
	0.3		—	0.0477	
	0.5		—	0.0083	
n-heptanol	0.1	No middle-phase microemulsion was formed	—	0.0696	—
	0.3		—	0.0220	
	0.5		—	0.0084	

**FIGURE 1:**
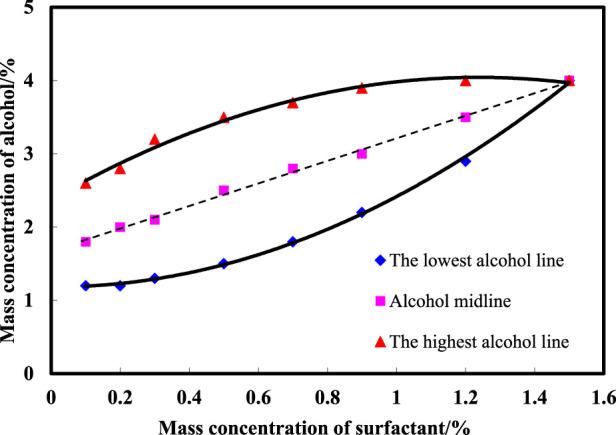
Microemulsion phase diagrams of different concentrations of surfactants with n-butanol.

Figure 1 illustrates the delineation of the middle-phase microemulsion boundary formed by varying amounts of n-butanol added to the surfactant compound system to ascertain the minimum quantities requisite for middle-phase microemulsion formation. By exploring the mass concentration range of surfactants from 0.1% to 1.5% and alcohol concentrations from 1.2% to 4.0%, observations were made on middle-phase microemulsion formation, identifying the lowest, midpoint, and highest alcohol points necessary for the middle phase. Fitting scatter data facilitated deriving the lines representing the lowest alcohol level, midpoint, and highest alcohol content. The graph demonstrates that as surfactant concentration increases, the alcohol width of the middle-phase microemulsion initially expands and then contracts. At a 0.5% surfactant concentration, the alcohol width range peaks at 2.0%. Subsequently, with surfactant concentrations surpassing 0.5%, the alcohol width range diminishes gradually. To optimize middle-phase microemulsion formation area maximization, the recommended n-butanol concentration falls within 1.3%–3.7%, alongside corresponding surfactant concentrations of 0.3%–0.7%.

### 3.2 Phase behavior of middle phase microemulsion

#### 3.2.1 Phase state of microemulsion under different salt concentration

**FIGURE 2:**
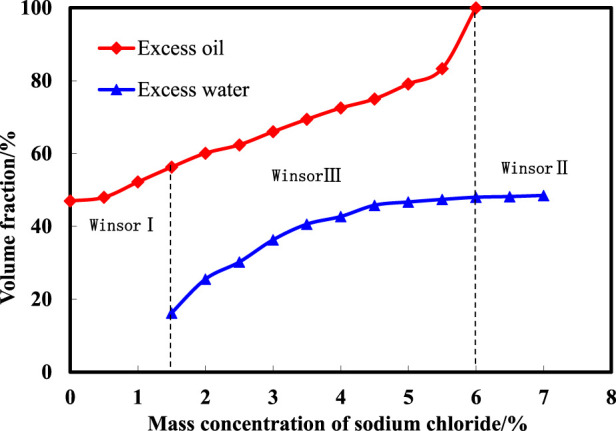
Microemulsion phase diagrams of different concentration of salinities.

The experimental process involved adding specific ratios of surfactant and alcohol to stopper colorimetric tubes, varying NaCl mass fractions, and combining equal volumes of crude oil and produced water. After sealing the tubes and shaking them vigorously, the solutions were left to stand at room temperature for 72 h. The stratification of the solution was then recorded, and the volume fractions of each phase (excess water, excess oil, and microemulsion) were measured to quantify the phases at different salinities. A Winsor phase diagram depicting salinity and phase volume relationships was generated. Figure 2 illustrates the results of salinity scanning in the microemulsion system. As NaCl concentration increases, the microemulsion system’s phase state transitions through Winsor I, Winsor III, and Winsor II phases sequentially. Salinity concentrations ranging from 0% to 1.5% exhibit a Winsor I phase (oil-in-water microemulsion). Within 1.5%–6.0% NaCl concentrations, a Winsor III phase emerges (bicontinuous phase microemulsion). Salinity concentrations exceeding 6.0% reveal a Winsor II phase (oil-in-water microemulsion). The salinity range associated with middle-phase formation and disappearance spans 1.5%–6.0%, with a 4.5% salt width facilitating middle-phase microemulsion formation.

#### 3.2.2 Solubilization ability under different salt concentrations

**FIGURE 3:**
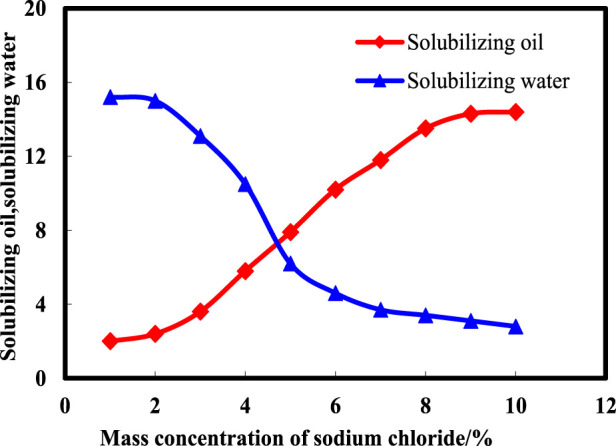
Solubilization capacity of middle-phase microemulsion at different salinities.

To determine the salinity conducive to optimal middle-phase microemulsion formation, a detailed subdivision of the salt width was undertaken alongside quantitative assessments of solubilization capacity. Solubilization ability serves as an indicator of surfactant interfacial activity, correlating stronger interfacial activity with heightened solubilization parameters and improved theoretical oil displacement efficacy. Formula 1 facilitated the calculation of solubilized oil and water quantities, aiding in the evaluation of surfactant performance. Figure 3 illustrates the variations in solubilized oil and water amounts at distinct NaCl mass concentrations within middle-phase microemulsions. Observations indicate a gradual decrease in solubilized water levels alongside an incremental increase in solubilized oil amounts as NaCl mass concentration rises. The salinity range of 4%–6% showcases enhanced solubilization effects, representing the optimal salinity window. At approximately 4.8% NaCl mass concentration, a critical point emerges where the quantities of solubilized oil and water become equivalent, signifying the theoretical optimal salinity value for achieving balanced solubility and efficient middle-phase microemulsion formation.

#### 3.2.3 Microemulsion interfacial tension

**FIGURE 4:**
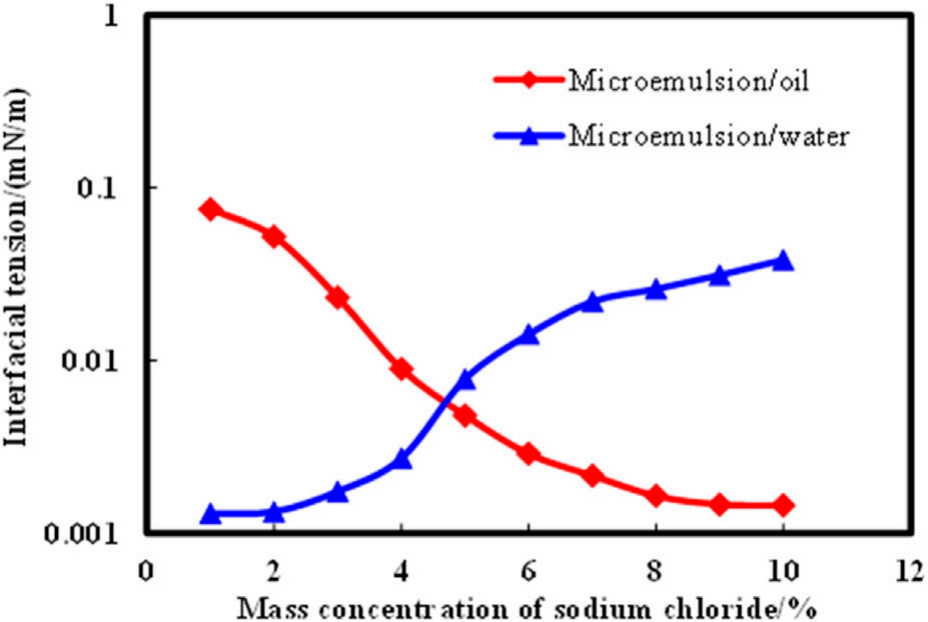
Effect of solubilization parameters on interfacial tension of microemulsion. (by calculation method).

**FIGURE 5:**
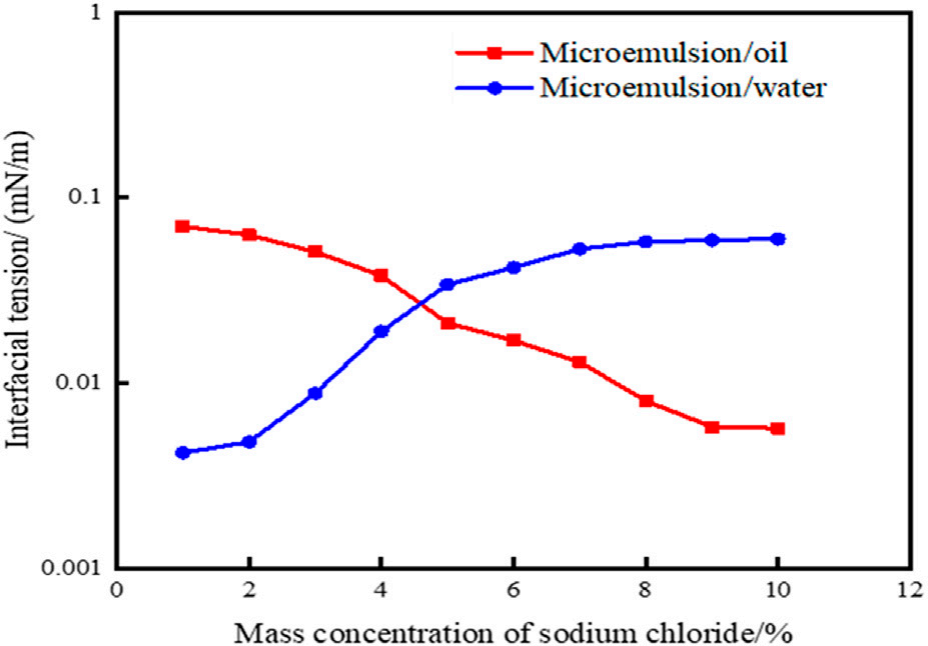
Effect of solubilization parameters on interfacial tension of microemulsion (by experimental method).

Formula 2 was utilized to compute the interfacial tensions of microemulsions across various NaCl mass concentrations, with outcomes depicted in Figure 4. Notably, as salinity levels ascend, the interfacial tension between the microemulsion and residual oil progressively diminishes, while the interfacial tension between the microemulsion and residual water gradually escalates. At NaCl mass concentrations exceeding 4.0%, the interfacial tension between the microemulsion and oil phase reaches an ultra-low level of 10^–3^ mN/m. Conversely, when NaCl mass concentrations fall below 5.0%, the interfacial tension between the microemulsion and aqueous phase achieves a 10^–3^ mN/m threshold. These trends underscore the critical role of salinity in modulating interfacial tensions within the microemulsion system, influencing its interactions with both oil and water phases. At the same time, the oil-water interfacial tension value of the microemulsion was obtained by the experimental measurement method. The experimental results are shown in Figure 5. From the comparison of Figures 4, 5, it can be seen that the error of the interface tension value obtained by the calculation method and the experimental method is within 10%, which verifies the accuracy and rationality of the calculation method.

### 3.3 Microscopic oil displacement mechanism of microemulsion

#### 3.3.1 Construction of microfluidic pore network model

**FIGURE 6:**
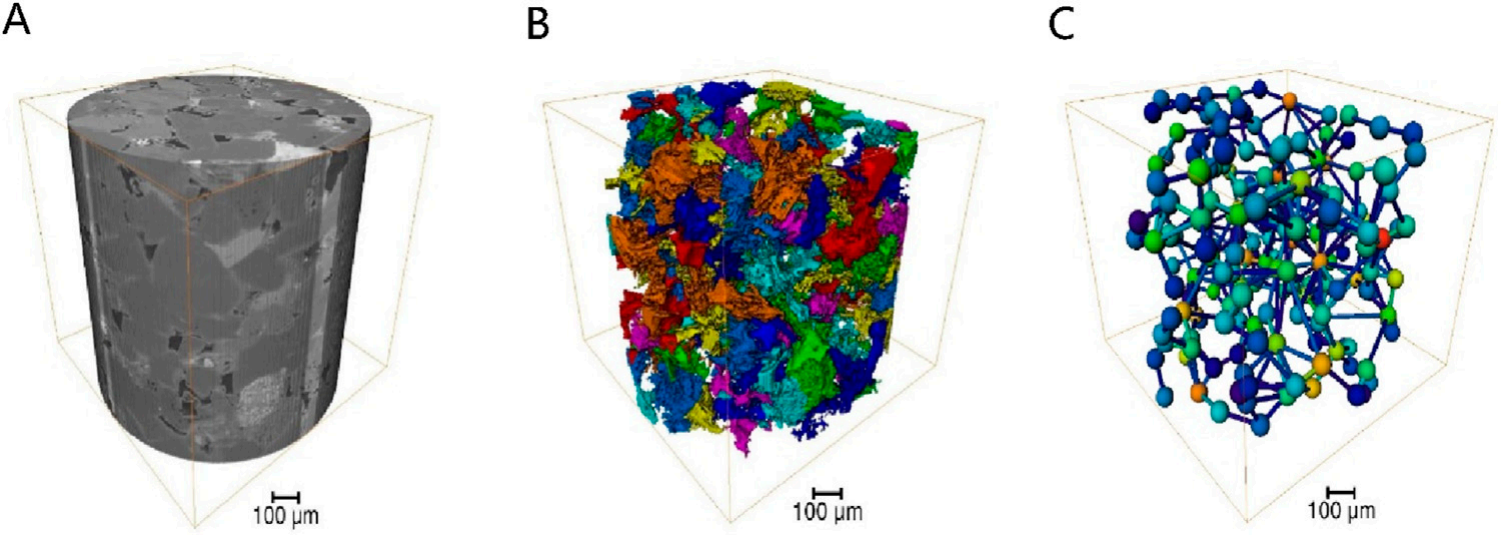
Three-dimensional digital core construction process: **(A)** three-dimensional image, **(B)** connectivity pores, **(C)** ball and stick model.

**FIGURE 7:**
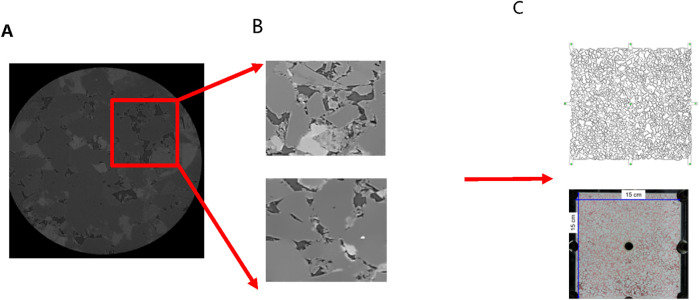
Procedures for designing and fabricating microfluidic chips: **(A)** Two-dimensional section slice, **(B)** typical pore structure, **(C)** large size reservoir chip.

The core of Changqing Oilfield was scanned using an X-ray micro-computed tomography scanner (Xradia 630 Versa, USA) to construct a high-precision three-dimensional digital core (Figure 6). By conducting two-dimensional core slicing and pore network numerical reconstruction, a microfluidic chip tailored for the target was fabricated using wet etching methods. Given the limitations of CT scanning field-of-view, a large-scale core image measuring 15 cm × 15 cm was generated through multiple scans and subsequent binarization processing, followed by image stitching. Utilizing OpenCV technology, down sampling and closed operation denoising procedures were implemented on the image. Subsequently, based on the chosen CT channel, the reconstructed pore network was exported, and AutoCAD software was employed to vectorize the pore network, culminating in the creation of the microfluidic chip as depicted in Figure 7.

#### 3.3.2 Microemulsion micro-displacement simulation

In microemulsion displacement experiments, diverse parameters such as injection volume, injection rate, concentration, and phase states were varied to explore the distribution of remaining oil and understand the mechanisms underlying different types of remaining oil. This comprehensive approach aimed to delineate how these factors influence the displacement process and subsequent oil recovery efficiency.

##### 3.3.2.1 Distribution type of remaining oil after water flooding

**FIGURE 8:**
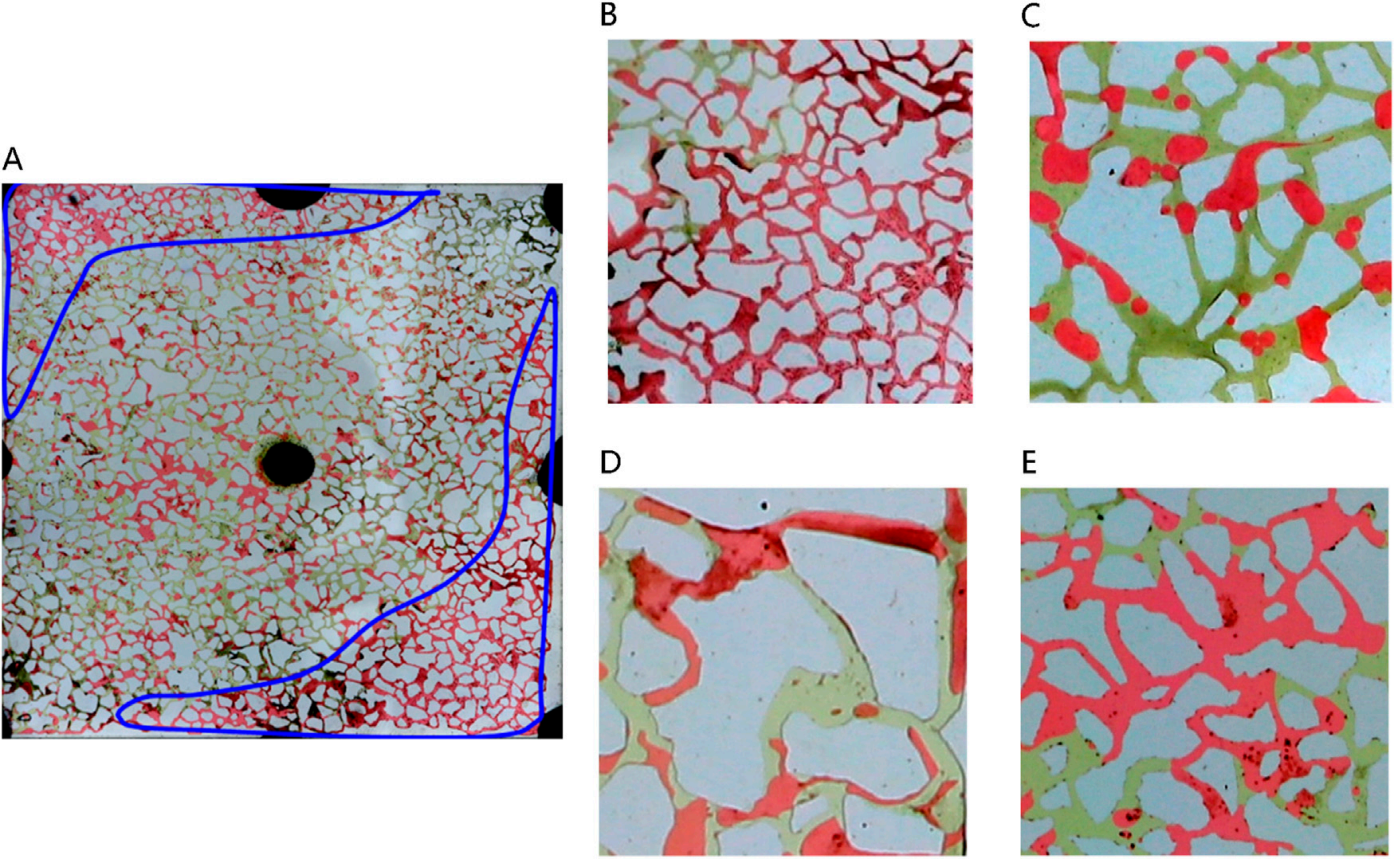
Classification of remaining oil morphology in water-wet wall reservoir chip: **(A)** Oil-water distribution after water flooding in large-scale reservoir chips. There are four kinds of typical residual oil: **(B)** unswept area, **(C)** oil droplets, **(D)** water film produced residual oil, **(E)** clusters. Red, yellow and white represent oil, water and solid, respectively.

Figure 8 depicts the various types of remaining oil distribution post water flooding. As water drive progresses to ultra-high water cut stages, limited oil mobility leads to diminished remaining oil within the mainstream channel; a significant portion of residual oil accumulates on either side of this channel (Figure 8A). Following water flooding, remaining oil distribution in pores is categorized into four main types: unswept area, oil droplets, water film protrusion, and cluster remaining oil (Figures 7B–E). In areas unswept by water on the sides of the mainstream region, pores are entirely filled with remaining oil. In contrast, at the core of the mainstream region, spherical oil droplets predominate due to the substantial interfacial tension between oil and water, impeding displacement during water flooding. These oil droplets, constrained by capillary forces, endure stably within the pores, posing challenges for expulsion. Furthermore, cluster oil and water film protrusions are also observed in the remaining oil within the pores. Cluster oil often forms large contiguous blocks, while water film protrusions adhere to pore walls. This distinction arises from the reservoir’s pronounced water-wet characteristics, where water films track along the wall, resulting in remaining oil either centralized within pores or adhering to a single wall surface.

##### 3.3.2.2 Characteristics of microemulsion flooding under different injection volume

**FIGURE 9:**
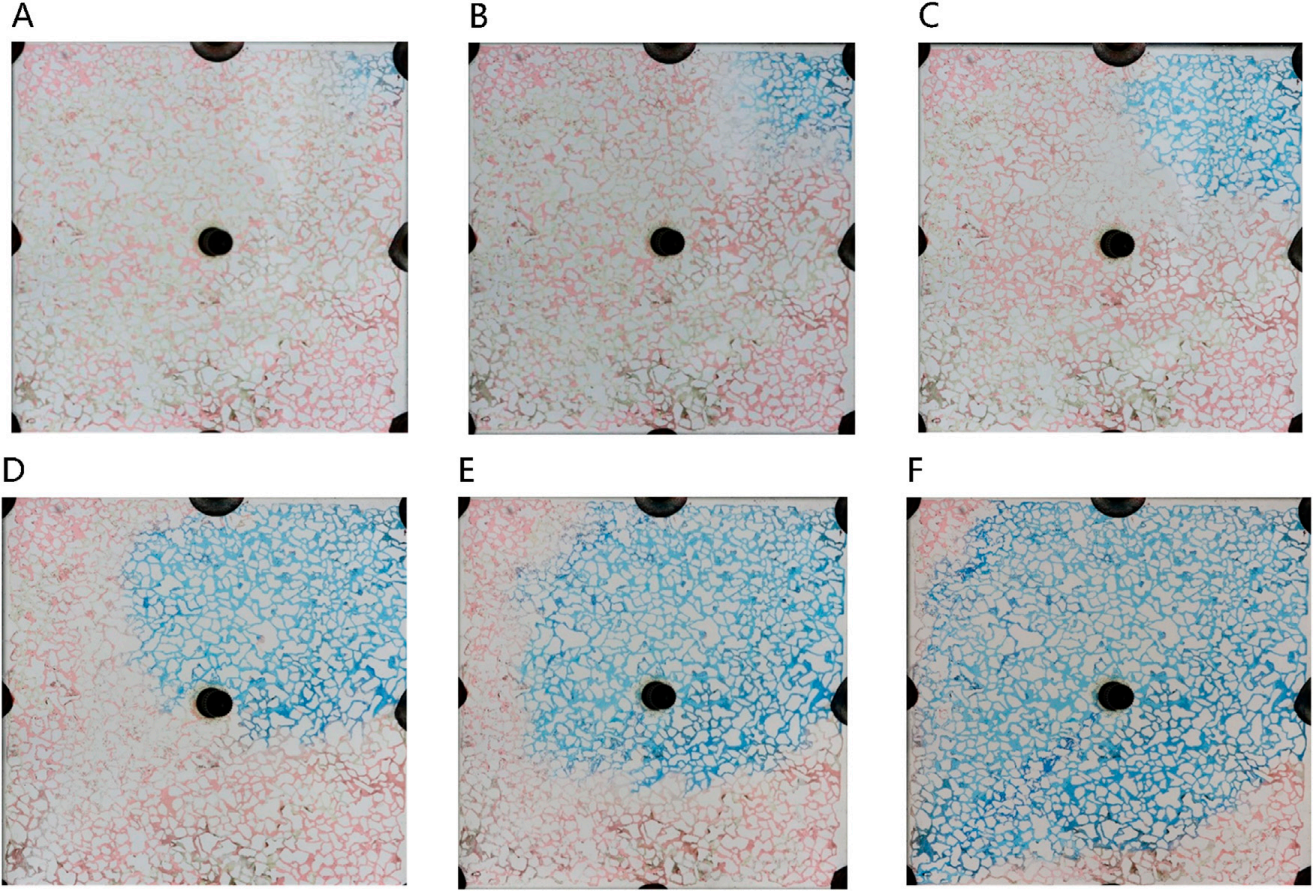
The sweep process of microemulsion flooding under different injection volumes: **(A)** 20 μL, **(B)** 80 μL, **(C)** 140 μL, **(D)** 200 μL, **(E)** 260 μL, **(F)** 320 μL. Red, blue and white represent oil, microemulsion system and solid, respectively.

The microemulsion was formulated using a 0.5% surfactant compound system, 3.5% n-butanol, and 4.8% NaCl concentration. It was subsequently injected at a consistent rate of 2 μL/min. Figure 9 illustrates the microscopic oil displacement mechanisms facilitated by the middle-phase microemulsion across varying injection volumes. This analysis provides insights into how different injection amounts affect the process of oil displacement at a microscale level.

**FIGURE 10:**
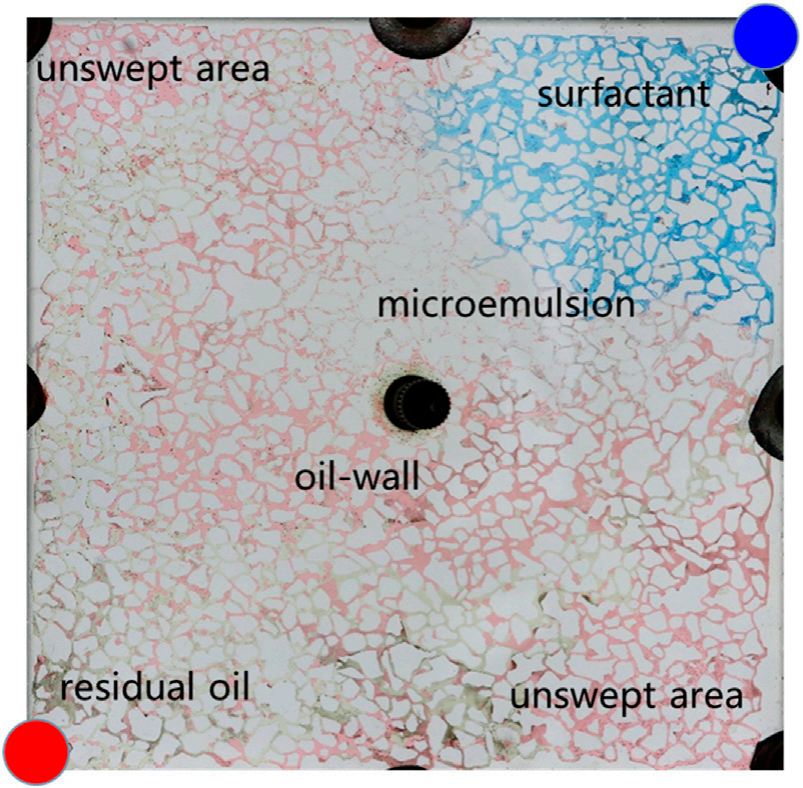
Oil and water distributions in the microfluidics during microemulsion flooding.

Figure 10 illustrates the spatial distribution characteristics of oil, water, and displacement fluid during the oil displacement process within a typical microemulsion system. The delineation includes distinct zones such as the surfactant area, microemulsion area, oil wall area, remaining oil area, and unswept area. Upon initial surfactant injection into the pore space, rapid mixing with the existing water occurs. In regions where the surfactant encounters residual oil at matching salinity levels, conditions foster swift formation of middle-phase microemulsion under kinetic constraints within the pore. This results in the emergence of a light-colored microemulsion area at the forefront of surfactant flooding. The *in-situ* generated middle-phase microemulsion exhibits high viscosity and establishes ultra-low interfacial tension with oil and water. Consequently, residual oil in the mainstream region is effectively utilized, leading to coalescence of oil droplets culminating in the creation of an oil wall at the displacement front. Areas untouched by the surfactant injection are categorized as the unswept area and remaining oil area. The former arises due to being situated on either side of the water flooding progression, while the latter manifests due to obstructions posed by the oil wall, configuring a water flooding procedure within the mainstream sector.

**FIGURE 11:**
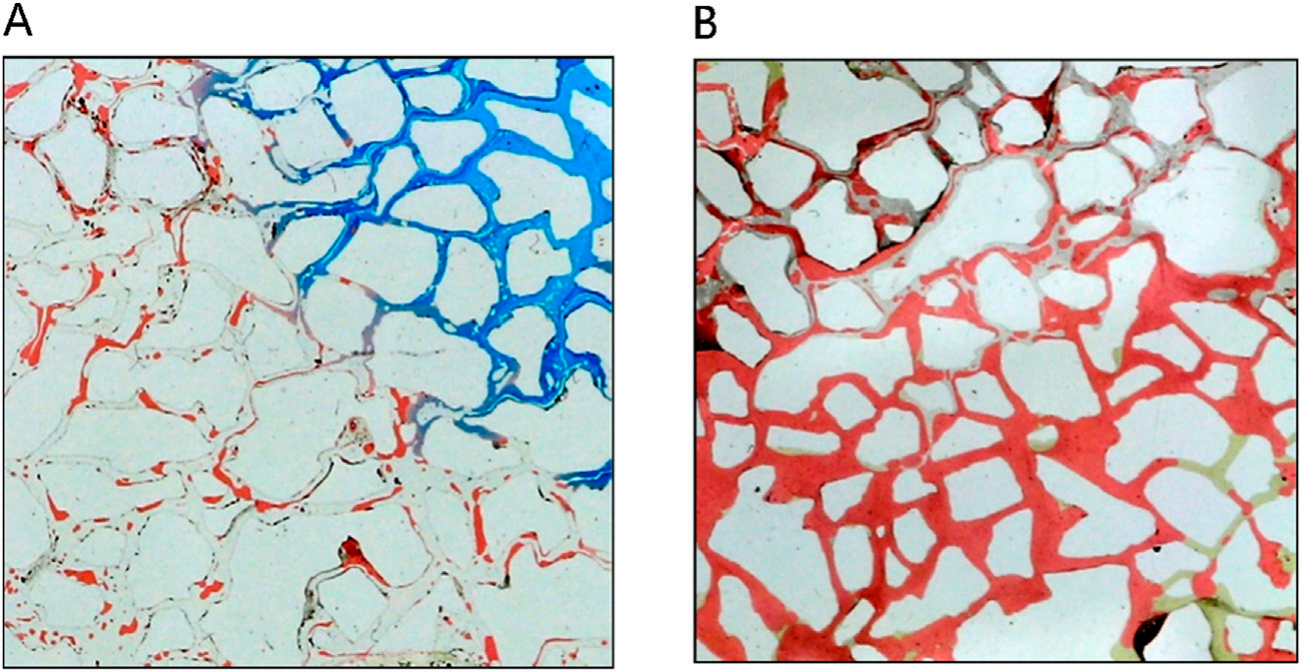
The fluid space distribution of microemulsion displacement: **(A)** Microemulsion zone, **(B)** Oil wall.

Figure 11 provides a detailed analysis of oil-water dynamics in distinct regions, focusing on the microemulsion region and the oil wall. In the microemulsion zone, oil droplets adopt a wire-like configuration, with water and oil blending within pores, resulting in a lighter coloration. The remaining oil within this segment exhibits hallmark ultra-low interfacial tension traits, appearing as minute oil droplets or wire-like structures. Conversely, within the oil wall area, remaining oil coalesces to form typical cluster oil distributions. This differentiation underscores how the characteristics and distribution of oil and water vary between the microemulsion region and the oil wall, offering insights into the complex fluid behavior during the displacement process.

In the analysis of the oil-water distribution patterns during middle-phase microemulsion displacement, several key observations emerge: (1) Upon surfactant introduction into the pore and interaction with oil/water interfaces, the nearby oil-water undergoes transformation into a solubilizing emulsion due to shear forces. (2) The resultant emulsion exhibits low interfacial tension with oil, prompting elongation of remaining oil under fluid shear, forming filaments that enhance oil-water contact area and emulsification. (3) The high viscosity of the formed emulsion impedes the advancement of the oil-water front, creating a high-viscosity microemulsion zone at the forefront of the water drive, facilitating spatial expansion. Downstream in the remaining oil region, unaffected by surfactant presence, water drive characteristics persist. An oil wall develops between the microemulsion and the remaining oil segment, delineating distinct fluid behavior zones within the displacement process.

**FIGURE 12:**
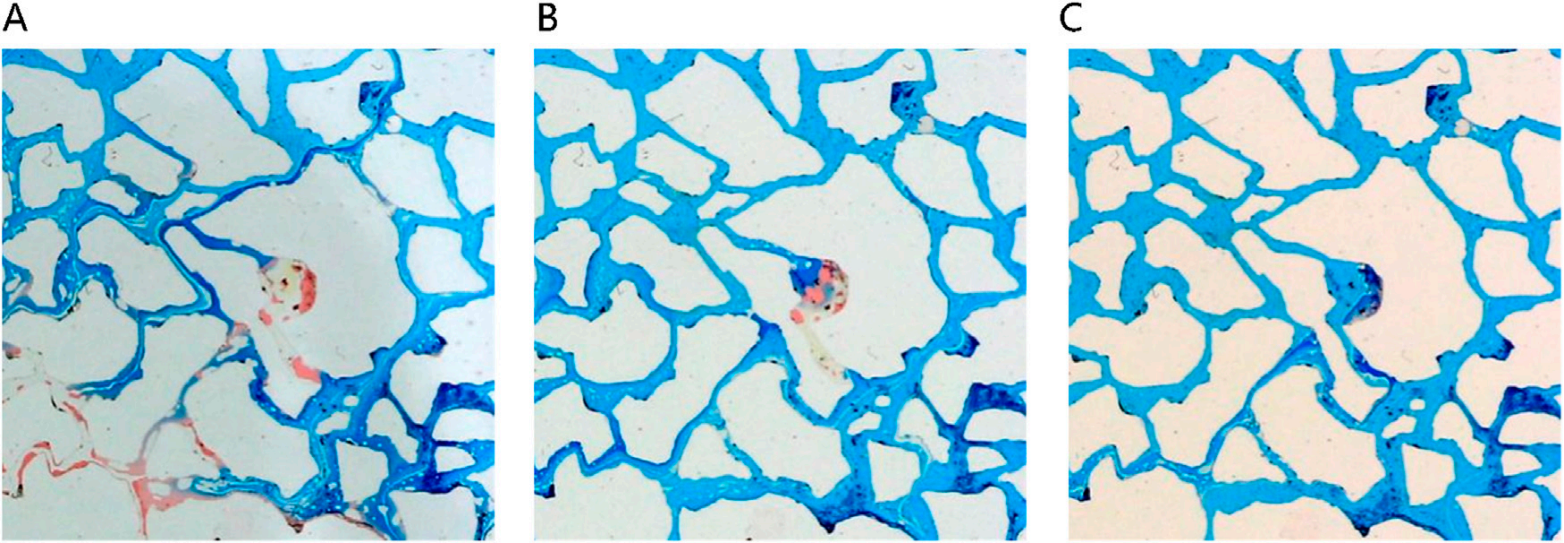
Blind end remaining oil displacing process under different injection volumes **(A)** 140 μL, **(B)** 180 μL, **(C)** 300 μL. Red, blue and white represent oil, microemulsion system and solid, respectively.

**FIGURE 13:**
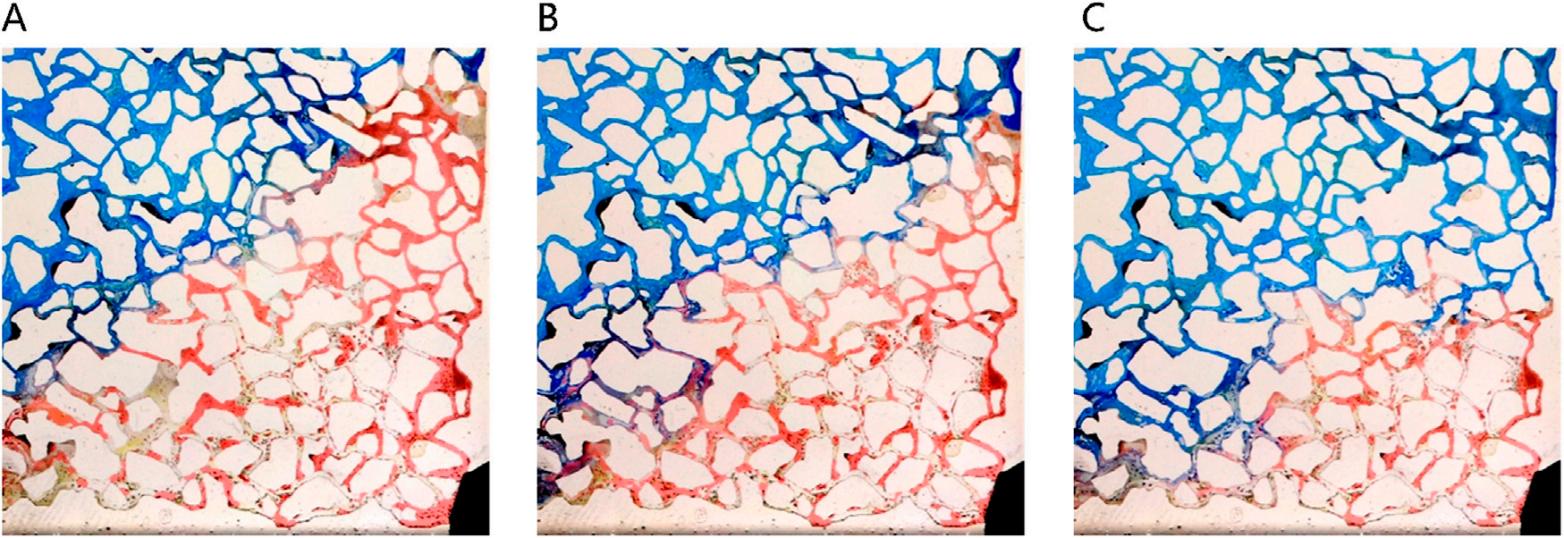
Remaining oil in unwept area displacing process under different injection volumes: **(A)** 300 μL, **(B)** 420 μL, **(C)** 540 μL. Red, blue and white represent oil, microemulsion system and solid, respectively.

Solubilization emerges as the primary mechanism driving the dissolution of blind-end residual oil by the middle-phase microemulsion, as depicted in Figure 12. However, hindered by water column blockages, surfactant diffusion towards the oil-water interface is gradual, impeding timely microemulsion formation due to weak shear effects. In contrast, for the unswept area, a swift outflow of fresh surfactant solution occurs through the mainstream channel, resulting in a comparatively slower utilization process within this sector. Figure 13 illustrates a gradual reduction in the red area of the unswept region and a relatively even distribution of the oil-water interface. Notably, the extensive red area on the right side signifies that residual oil utilization initiates from the outermost periphery, specifically at the oil-water interface; oil farther from this interface remains stationary. This observation underscores that unswept area residual oil exploitation primarily hinges on solubilization within the middle-phase microemulsion system rather than migration influenced by alternative mechanical conditions.

##### 3.3.2.3 Oil displacement characteristics of different concentrations of microemulsion

**FIGURE 14:**
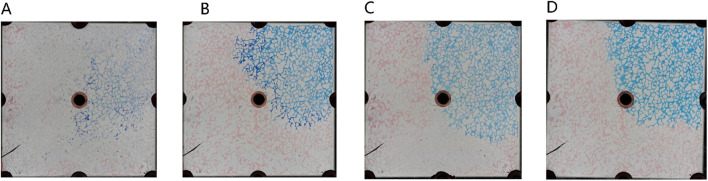
Sweeping characteristics of middle-phase microemulsion flooding at different concentrations: **(A)** The concentration of middle phase microemulsion is 0.5%, **(B)** The concentration of middle phase microemulsion is 1%, **(C)** The concentration of middle phase microemulsion is 4%, **(D)** The concentration of middle phase microemulsion is 8%. Red, blue and white represent oil, microemulsion system and solid, respectively.

Figure 14 illustrates the sweep characteristics of middle-phase microemulsion flooding at varied concentrations. The red area represents remaining oil, white denotes the solid skeleton, and blue signifies the middle-phase microemulsion system. Under low concentration conditions, limited emulsion formation hinders the creation of a distinct microemulsion band, leading to poor oil utilization as surfactant-containing water primarily flows along existing channels. Increasing surfactant concentration augments emulsion generation, enlarging the emulsion band, enhancing resistance against the oil-water front, stabilizing the water-driven advance, and boosting oil recovery efficiency. At extremely low surfactant concentrations, where middle-phase microemulsion formation is minimal or weak, no prominent microemulsion region emerges during displacement. Newly injected surfactant swiftly mixes with flooding water when surfactant concentration is near negligible, resulting in lighter-colored solutions due to thorough blending with water. This differentiation highlights how varying surfactant concentrations impact emulsion formation, displacement dynamics, and overall oil recovery efficacy in middle-phase microemulsion flooding processes.

**FIGURE 15:**
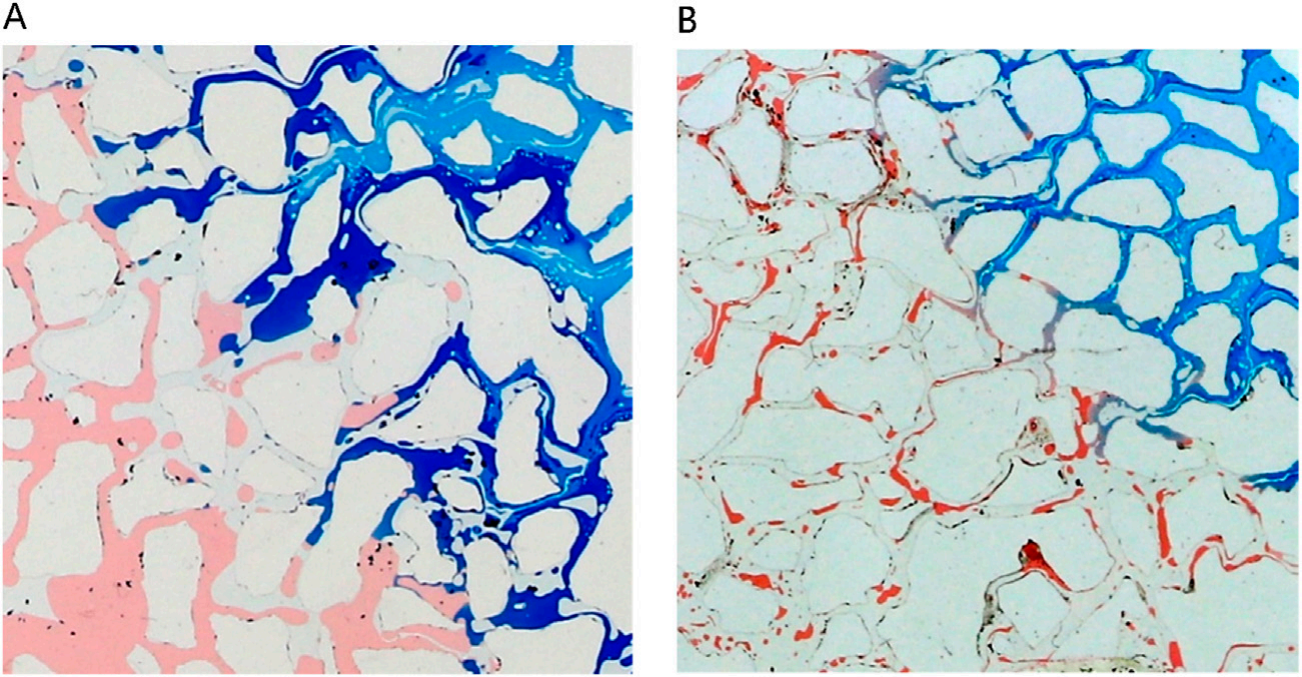
Microscopic mechanism of displacement front under different concentration conditions: **(A)** The concentration of middle phase microemulsion is 1%, **(B)** The concentration of middle phase microemulsion is 4%. Red, blue and white represent oil, microemulsion system and solid, respectively.

**FIGURE 16:**
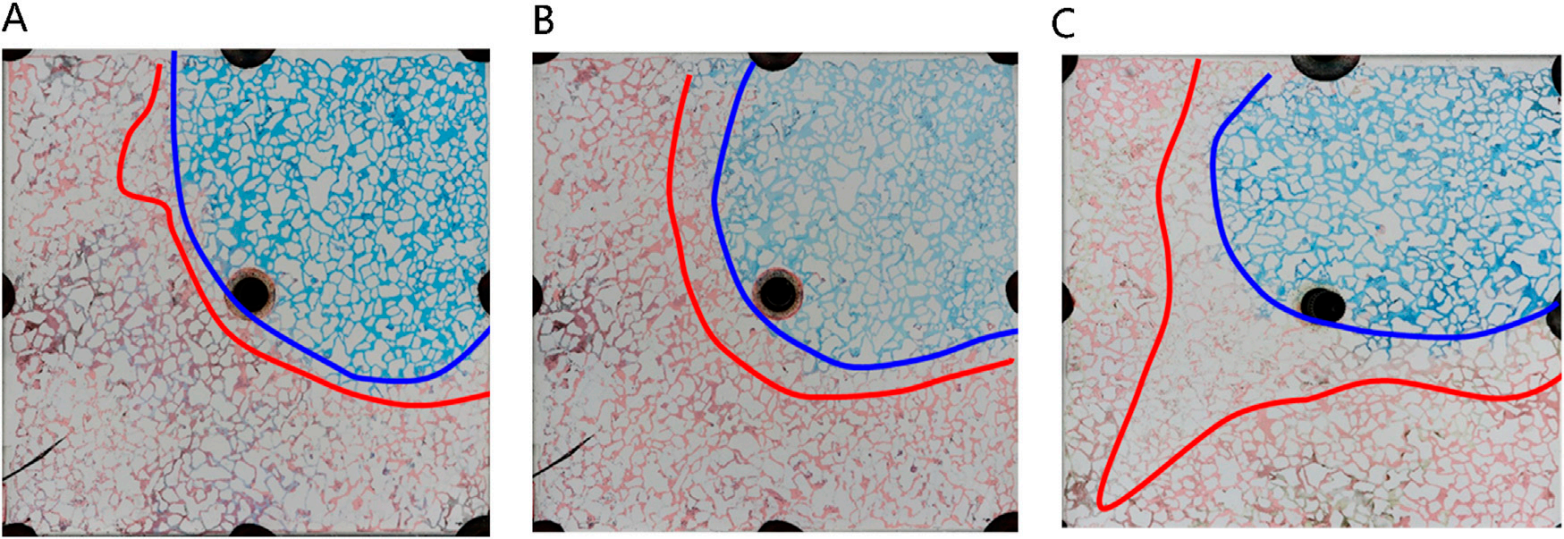
Microscopic oil displacement characteristics of microemulsion system under different phase states: **(A)** Winsor I (O/W) microemulsion, **(B)** Winsor II (W/O) microemulsion, **(C)** Winsor III (middle-phase) microemulsion. Red, blue and white represent oil, microemulsion system and solid, respectively.

Figure 15 dissects the microscopic displacement front mechanisms at varied concentration levels, specifically scrutinizing conditions set at 1% and 4%. Referring to Figure 16, it is evident that at low concentrations, residual oil retains its original form post-water flooding, with round oil droplets persisting. This preservation results from the substantial interfacial tension between the low-concentration middle-phase microemulsion and remaining oil, coupled with untransferred wettability. Conversely, at higher concentrations (particularly at 4%) rapid microemulsion formation occurs between the middle-phase microemulsion system and residual oil. Consequently, interfacial tension between oil and water sharply diminishes. Notably, alongside milky white microemulsion generation, substantial emulsified oil droplets within the remaining oil are prevalent, showcasing elongated characteristics. These augmented features are advantageous for maximizing the utilization of remaining oil, highlighting the efficacy of high-concentration microemulsion systems in enhancing oil recovery potential.

##### 3.3.2.4 Oil displacement characteristics of different phase microemulsion

In Figure 16, different phase states of microemulsions—oil-in-water, water-in-oil, and middle-phase—are employed to investigate residual characteristics post-water flooding. These microemulsions exhibit analogous oil-water distribution patterns, delineating regions such as the surfactant area, microemulsion area, oil wall area, remaining oil area, and unswept area. Upon initial surfactant injection into the pore, rapid mixing with pore water occurs. When encountering residual oil, microemulsion swiftly forms within the pore under kinetic conditions. Notably, the middle-phase microemulsion system boasts robust solubilization capabilities, resulting in a larger volume of formed microemulsion compared to other systems. This expanded volume is visually apparent in the diagram, demonstrating broader spatial coverage extending to the outlet under equivalent injection volumes. Conversely, water-in-oil or oil-in-water microemulsions typically manifest as thin leading edge layers at the forefront of surfactant flooding, contrasting with the more extensive spread and utilization facilitated by the middle-phase microemulsion system’s potent solubilization attributes.

**FIGURE 17:**
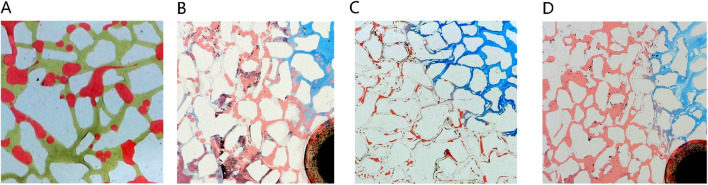
Morphology of residual oil: **(A)** after water flooding, **(B)** after Winsor I (O/W) microemulsion flooding, **(C)** after Winsor III (middle-phase) microemulsion flooding, **(D)** after Winsor II (W/O) microemulsion flooding. Red, yellow, blue and white represent oil, water, microemulsion system and solid, respectively.

Figure 17 presents a morphological contrast of remaining oil within the microemulsion system across different phase states and residual oil post-water flooding. After water flooding, residual oil primarily resides within pores in spherical droplet form. Elevated interfacial tension contributes to the spherical shape of remaining oil, concentrated mainly towards the rear of the pores. These oil droplets are relatively large, typically occupying individual pore sizes. In the case of oil-in-water microemulsion flooding, residual oil also appears as droplets; however, these droplets are not perfectly spherical, particularly evident at the forefront of surfactant flooding. Conversely, with middle-phase microemulsion flooding, remaining oil adopts a wire-like structure attributed to the ultra-low interfacial tension existing between the middle-phase microemulsion and oil. This wire-drawn configuration underscores how different phase state systems engender distinct morphological characteristics in residual oil post-flooding, illustrating the varying effects of interfacial interactions on oil distribution and shape within porous media.

## 4 Conclusion

In the context of Changqing Oilfield’s low-permeability reservoirs, a mixed surfactant system comprising sodium dodecyl benzene sulfonate and coconut oil fatty acid lipopolyoxyethylene betaine at a mass ratio of 1:3, alongside n-butanol as the cosurfactant, serves as the primary agent for middle-phase microemulsion development. The fish phase diagram guided the determination of an alcohol concentration range (1.3%–3.7%) essential for middle-phase microemulsion formation, with a corresponding surfactant system mass concentration of 0.3%–0.7%. The microemulsion exhibits a salt width of 4.5%, showcasing optimal solubilization and interfacial tension reduction to 10−3 mN/m at a NaCl mass concentration of 4.8%. Therefore, the medium-phase microemulsion system was composed of surfactant compound system, n-butanol and NaCl. The surfactant concentration was 0.5% (sodium dodecyl benzene sulfonate and coconut oil fatty acid lipopolyoxyethylene betaine at a mass ratio of 1:3), the concentration of n-butanol was 2.0%, and the concentration of NaCl was 4.8%.

For blind-end remaining oil utilization within the middle-phase microemulsion, solubilization emerges as the primary mechanism. Slow surfactant diffusion to the oil-water interface, coupled with weak shear effects hindering microemulsion formation, impacts efficiency. Similarly, in water flooding unswept areas, solubilization plays a key role, but swift mainstream channel outflow hampers unswept zone development. Under low concentration conditions, microemulsion zone formation is challenging, leading to limited crude oil utilization as surfactant-laden water primarily follows existing channels. Conversely, heightened surfactant concentrations augment emulsion generation, expanding the emulsion band, enhancing oil-water front hindrance, stabilizing water-driven fronts, and gradually boosting crude oil production capacity. The *in-situ* generated middle-phase microemulsion imparts viscosity augmentation compared to water flooding, fostering an oil wall at the displacement forefront. This promotes residual oil accumulation and movement, thereby bolstering oil recovery efforts. Ultra-low interfacial tension between middle-phase microemulsion and crude oil causes residual oil to elongate and fracture into small droplets, facilitating passage through micro-pore throats and displacement pathways.

From the visualization experiment using the large-scale microfluidic chip, it can be seen that the middle-phase microemulsion system proposed in this study can effectively recovery the remaining oil in the pores of low-permeability porous media. Although the pore structure of microfluidic chips is extracted from real rock cores, some necessary simplifications have been made. Therefore, it is necessary to further study in real rock cores and gradually promote it to field experiments.

## Data Availability

The raw data supporting the conclusions of this article will be made available by the authors, without undue reservation.
